# Anti-*Streptococcus mutans* and anti-biofilm activities of dextranase and its encapsulation in alginate beads for application in toothpaste

**DOI:** 10.7717/peerj.10165

**Published:** 2020-11-17

**Authors:** Nucharee Juntarachot, Sasithorn Sirilun, Duangporn Kantachote, Phakkharawat Sittiprapaporn, Piyachat Tongpong, Sartjin Peerajan, Chaiyavat Chaiyasut

**Affiliations:** 1 Innovation Center for Holistic Health, Nutraceuticals and Cosmeceuticals, Faculty of Pharmacy, Chiang Mai University, Mueang Chiang Mai, Chiang Mai, Thailand; 2 Department of Microbiology, Faculty of Science, Prince of Songkla University, Hat Yai, Songkhla, Thailand; 3 Brain Science and Engineering Innovation Research Group, School of Anti-Aging and Regenerative Medicine and Department of Anti-Aging Science, School of Anti-Aging and Regenerative Medicine, Mae Fah Luang University, Wattana, Bangkok, Thailand; 4 Health Innovation Institute, Mueang Chiang Mai, Chiang Mai, Thailand

**Keywords:** *Streptococcus mutans*, Dextranase, Encapsulation, Oral care, Toothpaste, Cariogenic bacteria

## Abstract

**Background:**

The accumulation of plaque causes oral diseases. Dental plaque is formed on teeth surfaces by oral bacterial pathogens, particularly *Streptococcus mutans*, in the oral cavity. Dextranase is one of the enzymes involved in antiplaque accumulation as it can prevent dental caries by the degradation of dextran, which is a component of plaque biofilm. This led to the idea of creating toothpaste containing dextranase for preventing oral diseases. However, the dextranase enzyme must be stable in the product; therefore, encapsulation is an attractive way to increase the stability of this enzyme.

**Methods:**

The activity of food-grade fungal dextranase was measured on the basis of increasing ratio of reducing sugar concentration, determined by the reaction with 3, 5-dinitrosalicylic acid reagent. The efficiency of the dextranase enzyme was investigated based on its minimal inhibitory concentration (MIC) against biofilm formation by *S. mutans* ATCC 25175. Box-Behnken design (BBD) was used to study the three factors affecting encapsulation: pH, calcium chloride concentration, and sodium alginate concentration. Encapsulation efficiency (% EE) and the activity of dextranase enzyme trapped in alginate beads were determined. Then, the encapsulated dextranase in alginate beads was added to toothpaste base, and the stability of the enzyme was examined. Finally, sensory test and safety evaluation of toothpaste containing encapsulated dextranase were done.

**Results:**

The highest activity of the dextranase enzyme was 4401.71 unit/g at a pH of 6 and 37 °C. The dextranase at its MIC (4.5 unit/g) showed strong inhibition against the growth of *S. mutans*. This enzyme at 1/2 MIC also showed a remarkable decrease in biofilm formation by *S. mutans*. The most effective condition of dextranase encapsulation was at a pH of 7, 20% w/v calcium chloride and 0.85% w/v sodium alginate. Toothpaste containing encapsulated dextranase alginate beads produced under suitable condition was stable after 3 months of storage, while the sensory test of the product was accepted at level 3 (like slightly), and it was safe.

**Conclusion:**

This research achieved an alternative health product for oral care by formulating toothpaste with dextranase encapsulated in effective alginate beads to act against cariogenic bacteria, like *S. mutants*, by preventing dental plaque.

## Introduction

Dextranase is an enzyme that catalyzes the endohydrolysis of *α*-(1–6)-D-glycoside linkages in dextran (Wang et al., 2014). The dental plaque or biofilm in the oral cavity consists of dextran, a product of dental caries pathogens, especially *Streptococcus mutans* (Forssten, Björklund & Ouwehand, 2010). Previous reports showed that dextranase breaks down the structure of biofilm (Schachtele, Staat & Harlander, 1975; Morel Du Boil & Wienese, 2002; Ren et al., 2018). In recent years, dextranase has received considerable attention in the food, medicine, and dental fields (Wang et al., 2018). However, there are some limitations to using this enzyme on the industrial scale. For example, dextranase has poor stability in harsh environments, which causes adverse effects on enzyme activity (Wang et al., 2018). The causes of instability of enzymes in oral care products are many, including temperature, acid, base, surfactant, and other composition of oral care products (Mateo et al., 2007). Therefore, it is highly desirable to develop effective methods for increasing the stability of dextranase (Wang et al., 2018).

Encapsulation is commonly used to protect active compounds against adverse environmental and processing conditions or to provide controlled release in processed foods (Kim et al., 2019). After encapsulation, the active compounds are significantly improved for potential applications as active packaging (Niu et al., 2020). There have been previous reports of encapsulation of calcium and phosphate ions in a toothpaste formulation, which could significantly release the active compounds while using the toothbrush for enhanced re-mineralization (Cooper, 2013). Thus, it is possible to encapsulate enzymes for application in industrial and cosmetic products (Wang et al., 2014). The encapsulation of important substances using food-grade matrices, like alginate, is a suitable procedure to preserve their stability. It protects the surface of the substance from virulent environments and prevents damage. Sodium alginate is one of the most widely investigated ones in the pharmaceutical and biomedical fields, and its monograph is included in both the European Pharmacopeia and the United States Pharmacopeia (Szekalska et al., 2016). The calcium reactivity of alginate is a consequence of the particular molecular geometries of each region. Sodium alginate is capable of forming rigid gels by the action of calcium ion or multivalent cations (Sharma et al., 2018). Optimization of encapsulation for protecting dextranase is one of the most important stages. Response surface methodology (RSM) is an effective optimization tool with which many factors and their interactions can be identified with fewer experimental trials (Pasma et al., 2013). Placket Burman design is popularly used to design for find out which factors in an experiment are important (Wasko et al., 2010). While, the Box-Behnken design (BBD) is normally employed to investigate the optimal conditions as a requirement of minimal experimental number compared with other designs (Tafreshi, Sharifnia & Moradi Dehaghi, 2017).

The use of antimicrobials as an important adjunct to tooth brushing may also play an important role in controlling biofilm formation. Recently, the incorporation of chemical agents with antimicrobial activity into dental products has been proposed as a potential way of reducing plaque-mediated disease (Amižić et al., 2017). The possible action of toothpaste containing dextranase enzyme in the oral cavity is the destruction of biofilm, which causes dental caries. This is a new concept in the prevention of oral infectious diseases, such as caries.

Regarding the above information, this research aims to determine the most effective condition for the encapsulation of dextranase enzyme using response surface methodology. The suitable condition of encapsulation for trapping dextranase was determined for the possibility of applying it in toothpaste. Finally, the acceptability of toothpaste containing encapsulated dextranase enzyme in alginate beads was evaluated based on safety and satisfaction; this could serve as a reference in the formulation and development of toothpaste containing dextranase in the future.

## Materials & Methods

### Measurement of dextranase activity

Dextranase can be produced by various microorganisms, including bacteria, yeasts, and filamentous fungi. However, our preliminary results found that fungal dextranase is more effective in degrading dextran than those of other microbes. Hence, the fungal commercial dextranase was selected for use in this study, and it was produced from *Chaetomium gracile*; dextranase from *C. gracile* is generally recognized as safe (GRAS) (Virgen-Ortíz et al., 2015) for use in foods. This confirms its safety; therefore, food-grade dextranase was purchased from Mitsubishi chemical foods corporation, Japan. Dextranase activity was measured based on an increasing ratio of the reducing sugar concentration in the reaction with 3,5-dinitrosalicylic acid reagent (Wang et al., 2014). A mixture of 200 mg/mL of commercial dextranase (125 µL) and 20 mg/mL of dextran solution (125 µL) was incubated at 37 °C for 30 min. The reaction was stopped after 30 min by transferring 250 µL aliquots of the enzyme-substrate mix into tubes containing 3,5-dinitrosalicylic acid reagent. Then, the tubes were incubated for 15 min in a boiling water bath, and two mL of distilled water was added. The absorbance of the mixture was measured at 540 nm. One unit of dextranase activity (U) was defined as the amount of enzyme that catalyzed the liberation of one mmol of maltose in 1 min from dextran. In order to assess the possibility of adding commercial dextranase to toothpaste to prevent dental caries, the optimal condition of this enzyme was investigated by varying the pH (3, 4, 5, 6 and 7) and temperature (25, 37, 45 and 55 °C).

### Minimal inhibitory concentration (MIC)

The MIC of dextranase against the growth of *S. mutans* ATCC 25175 was investigated by the broth dilution method. Briefly, *S. mutans* grown in Tryptic Soy Broth (TSB) was adjusted by the normal saline solution to obtain 10^6^ cells/mL, and then mixed with the serially diluted test of dextranase (144–0.07 unit/g concentrations) in a 96 well-plate, followed by incubation at 37 °C, 5% CO_2_ for 24 h. The MIC was determined by spectrophotometric analysis of the growth of *S. mutans* at 600 nm (Thenmozhi et al., 2009).

### Effect of dextranase on biofilm formation of *Streptococcus mutans*

The effect of sub-MIC (1/2 MIC) of dextranase against biofilm-formation by *S. mutans* was tested with the modification described by Sato et al. (2018); 100 µL of 3.38 unit/g dextranase enzyme was added into 100 µL of TSB broth containing bacterial suspension at 10^6^ CFU/ml. The 96 well plates were incubated for 24 h at 37 °C and 5% CO_2_. After incubation, the biofilm was stained with 0.4% crystal violet for 15 min. The cells were then washed three times with sterile distilled water and air-dried for 60 min. Stained biofilm cells were de-stained using 95% ethanol. Biofilm removal was determined on the basis of visible disruption in biofilm formation and a significant reduction in the readings at OD_570_ nm by comparison with the negative control wells with no *S. mutans* (Thenmozhi et al., 2009). *S. mutans* in TSB broth without the addition of dextranase served as the positive control (Table 1). The dextranase enzyme was classified into the following categories based on the OD of bacterial films: non-adherent, weak, moderate, and strong adherent. The cut-off OD for the microtiter-plate test is defined as three standard deviations above the mean OD of the negative control by following the classification according to Stepanović et al. (2000). All tests were carried out three different times and the results were averaged.

OD ≤ ODc (OD in negative control) (non-adherent)

ODc <OD ≤ 2ODc (weakly adherent)

2ODc <OD ≤ 4ODc (moderately adherent)

4ODc <OD (strongly adherent)

### Anti-biofilm assay: Confocal Laser Scanning Microscopy (CLSM)

Confocal laser scanning microscopy (CLSM) was used to determine the anti-biofilm activity of dextranase. *S. mutans* ATCC 25175 was used as a biofilm producer in this study. The experiment was a modification of broth dilution method (Thenmozhi et al., 2009); *S. mutans* was grown in TSB and adjusted to 10^6^ cells/mL; then, one mL of *S. mutans* cell suspension was mixed with one mL of sub-MIC (1/2 MIC) dextranase. The biofilms were allowed to grow on 1 cm ×1 cm glass slide placed in 24-well titer plates, followed by incubation at 37 °C and 5% CO_2_. After 24 h incubation, the biofilm formed was stained with SYTO^®^9 green fluorescent dye (Sigma). The samples were assessed by Nikon Laser Confocal Microscope C1. Images were captured and processed using EZ-C1 version 3.90. The results were compared with the control as without dextranase addition.

**Table 1:** The effect of commercial dextranase on biofilm adherent capability of *Streptococcus mutans*.

**Treatment**	**OD**_**570**_**nm**		**Adherent capability**
	**0 h**	**24 h**	
Uninoculated medium (negative control)	0.24 ± 0.00	0.24 ± 0.00	Non-adherent
*S . mutans* (positive control)	0.25 ± 0.01	0.98 ± 0.01	Strongly adherent
Commercial dextranase (1/2 MIC)	0.24 ± 0.00	0.47 ± 0.01	Weakly adherent

**Notes.**

All values provided as mean ± standard deviations of triplicate.

### Encapsulation of dextranase enzyme: The study of factors affecting encapsulation to achieve a suitable condition

On the basis of our preliminary work, the independent variables were firstly assessed by Placket Burman design. pH, alginate and calcium chloride concentrations were found as the significant factors on encapsulation efficiency (% EE) and dextranase activity (Dataset S1). Therefore, these three factors were selected as the independent variables tested in the experiment of the Box-Behnken design experiment for achievement of optimal conditions on % EE and dextranase activity as the responses for the combination of the independent variables. Box-Behnken design (BBD) was used to study the three factors affecting encapsulation: pH, calcium chloride concentration and the concentration of sodium alginate. Each of these variables was studied at three different levels (−1, 0, 1) using BBD. The encapsulation of commercial dextranase was designed to have 17 conditions. Dextranase enzyme solution (concentration: 20 mg/ml) and sodium alginate solution (concentration: 0.7%, 0.85%, 1.0%) were mixed at a ratio of 1:1, and the pH of the mixed solution was adjusted (pH: 5, 6, 7). The software package Design Expert, version 10.0 (Stat-Ease Inc., Minneapolis, MN, USA) was used for the experimental design, data analysis and model building. According to analysis of variances (ANOVA) which was applied to assess effects of studied variables, interactions and statistical significance of models) the fitness of the polynomial model equations were expressed by the coefficient of determination R^2^ (Peng et al., 2020).

Three-dimensional response surface plots were drawn to identify the interaction between factors and responses. The Buchi encapsulator was used to prepare the capsules. The nozzle type and size used were single 450 µm, 160 Hz of frequency, 11–15 ml/min of flow rate, 500–600 V of the electrode, 100–150 m Bar of air pressure and 30 min of hardening time. The capsules were dropped into the calcium chloride solution (concentration 10%, 15%, 20%), and the beads were filtered and washed with distilled water 2 times to eliminate excess calcium chloride (Bashari et al., 2013). The characteristics and mean diameter of beads were studied by ZEN lite imaging software of Stereo microscopes (ZEISS Stemi 305).

### Encapsulation efficiency (% EE): Protein content in the dextranase enzyme solution and sodium alginate

A quantity of 20 µL of the mixed solution of dextranase enzyme solution (concentration: 20 mg/ml) and sodium alginate (concentration: 0.7%, 0.85%, 1.0%) at a ratio of 1:1 was placed in a microplate. Afterward, 200 µL of Bradford reagent was added into the microplate, and the absorbance of the reaction solution was measured using the UV spectrophotometer at a wavelength of 595 nm. The amount of protein in the mixed solution was calculated (Bashari et al., 2013).

### Encapsulation efficiency (% EE): Protein content in calcium chloride solution

A quantity of 20 µL of calcium chloride solution after the encapsulation process was placed in a microplate, followed by the addition of 200 µL of Bradford reagent. The absorbance of the reaction solution was measured using the UV spectrophotometer at a wavelength of 595 nm. The amount of protein in the mixed solution was calculated (Bashari et al., 2013). Also, the encapsulation efficiency was calculated as follows.

\begin{eqnarray*}\text{%}~\text{Encapsulation efficiency}=((A-B)\times 100)/A \end{eqnarray*}

A = The amount of protein from encapsulation

B = Protein content in calcium chloride solution.

### The activity of encapsulated dextranase enzyme in alginate beads

One gram (1g) of beads was grained with 1 g of 0.1 M phosphate buffer saline (PBS, pH 6.0), and 125 µL of bead solution was mixed with 125 µL of dextran solution for 30 min. A quantity of 250 µL of DNS reagent was pipetted into a test tube, and the test tube was immersed in a boiling water bath for 15 min. Then, the test tube was soaked in cool water, and two mL of distilled water was added to dilute the color. A quantity of 200 µL of the reaction solution was pipetted into a microplate. The absorbance was measured with a UV spectrophotometer at a wavelength of 540 nm. The activity of dextranase was calculated as described by Bashari et al. (2013).

### The stability of encapsulated dextranase activity in a toothpaste base

The toothpaste base is a composite of humectant, preservative, sweetening agent, abrasive, coloring agent, and detergent (Grace et al., 2015). As the MIC of dextranase against the growth of *S. mutans* was 4.50 units/g, the concentration of dextranase was varied by increasing the MIC 10 times, 20 times, and 30 times. These were equivalent to 1%, 2% and 3% alginate beads in the formulas for mixing with toothpaste. It was found that the formulation containing 2% beads with dextranase showed good physical characteristic (Table S1). Besides, the dose selection was considered based on the range of human equivalent dose (HED) for clinical trial safety. Then, the dextranase encapsulation at 2% w/w alginate beads was added to the toothpaste base. The storage experiment followed the guideline of the Thai industrial standards (TIS 45-2552-2009 Toothpastes). Hence, the toothpaste was exposed to accelerated storage condition at 40 ± 2 °C for three months to check the stability of the encapsulated dextranase activity in the toothpaste. Dextranase activity was measured as previously described.

### The sensory test

This research focused on the efficiency of anti-biofilm to prevent tooth decay. However, safety is very important to be considered for dental care products, while sensory acceptance by consumers is also important for the possibility to be able to compete in the market. Therefore, the evaluation of the safety and satisfaction of the product is important to gain the basis for improvement and development of toothpaste product. This experiment is a pilot study to assess the satisfaction of toothpaste containing dextranase commercial enzyme encapsulated in alginate. The satisfaction and safety of developed toothpaste was evaluated by 15 volunteers based on the modification of the guidelines of Chowdhury et al. (2013). The ethic of volunteering was approved by the Research Ethics Committee, Faculty of Pharmacy, Chiang Mai University, Thailand with the ethic approval number 05/2562. The research was also submitted to the Thai Clinical Trials Registry (TCTR), a research database for clinical research of Thailand with a study ID: TCTR20200409003 (http://www.clinicaltrials.in.th/).

### Inclusion criteria

1. Female or male volunteers aged 20 years or more.

2. Volunteers who agree to participate in the research.

### Exclusion criteria

1. Volunteers during pregnancy.

2. People who have a history of cosmetic allergy.

3. People with oral ulcers, those who have taken out teeth or have undergone oral surgery.

### Pre-test: oral assessment in volunteers

To make sure that volunteers do not have oral ulcers, their oral cavities were evaluated by a dentist. The result of the evaluation was recorded by the dentist.

### Test: volunteers brush their teeth with toothpaste

The volunteers were given toothpaste containing dextranase enzyme and brushed their teeth with the toothpaste for 2 min. They were evaluated during and after brushing.

### Post-test: oral assessment in volunteers

To make sure that volunteers do not have irritation after brushing, their oral cavities were evaluated by a dentist. The result of the evaluation was recorded by the dentist (Wattanakrai et al., 2007).

### Final-test: assess satisfaction with volunteers

The acceptability of the toothpaste containing encapsulated commercial dextranase was assessed by sensory analysis. Several parameters, including color, odor, flavor, distribution, homogeneity, foaming, clean feeling after use, fresh feeling after use and overall satisfaction, were measured. A 5-point hedonic scale was used to assess the suitability (Graham, Agbenorhevi & Kpodo, 2017).

## Results

### Measurement of dextranase activity

The activity of commercial dextranase was examined for its optimal pH. The highest dextranase enzyme activity, 4155.09 unit/g, was observed at a pH of 6, followed by pH of 5 and 7, while the minimum activity was observed at pH of 3–4, roughly 1,000 unit/g. The optimal temperature of commercial dextranase activity under the optimal pH of 6 was between 25 and 37 °C with the highest activity of 4,401.71 unit /g, followed by 45 °C. A huge decrease was observed at 55 °C with only 1000 unit/g. Hence, the optimum condition of this enzyme, pH of 6 and 37 °C, was selected to measure its activity in all experiments of this research (Table S2).

### Minimal inhibitory concentration (MIC)

The MIC assay is a technique used to determine the lowest concentration of treatments needed to inhibit bacteria. Inhibition was observed after incubation at 37 °C for 24 h. The MIC was read as the lowest concentration of the dextranase enzyme at which there is no visible growth. In determining the OD_600_ values, the dextranase showed positive antibacterial activity against the growth of *S. mutans*, and its MIC was 4.5 unit/g. This was done along with the determination of the MIC of the positive control (penicillin G) against *S. mutans*. The MIC observed for penicillin G was 0.24 µg/ml (Table S3).

### Effect of dextranase on biofilm formation of *S. mutans*

An experiment was performed to measure the adherence capacity of the biofilm formed by *S. mutans.* To confirm the efficacy of dextranase enzyme regarding biofilm removal, as it is one of the ways to prevent dental plaque, the biofilm was dyed with 0.4% crystal violet, and the absorbance was measured with at a wavelength of 570 nm. Biofilm removal was determined based on visible disruption in biofilm formation in the readings compared with the control wells. The summarized results of the microtiter plate tests are presented in Table 1 and Table S4. The medium without *S. mutans* served as the negative control (ODc = 0.24), which showed non-adherence. The dextranase enzyme showed ODc <0.47 ≤ 2ODc, which was classified as weakly adherent, while the positive control with only *S. mutans* without dextranase showed strong-adherence.

### Anti-biofilm assay: Confocal Laser Scanning Microscopy (CLSM)

**Figure 1:**
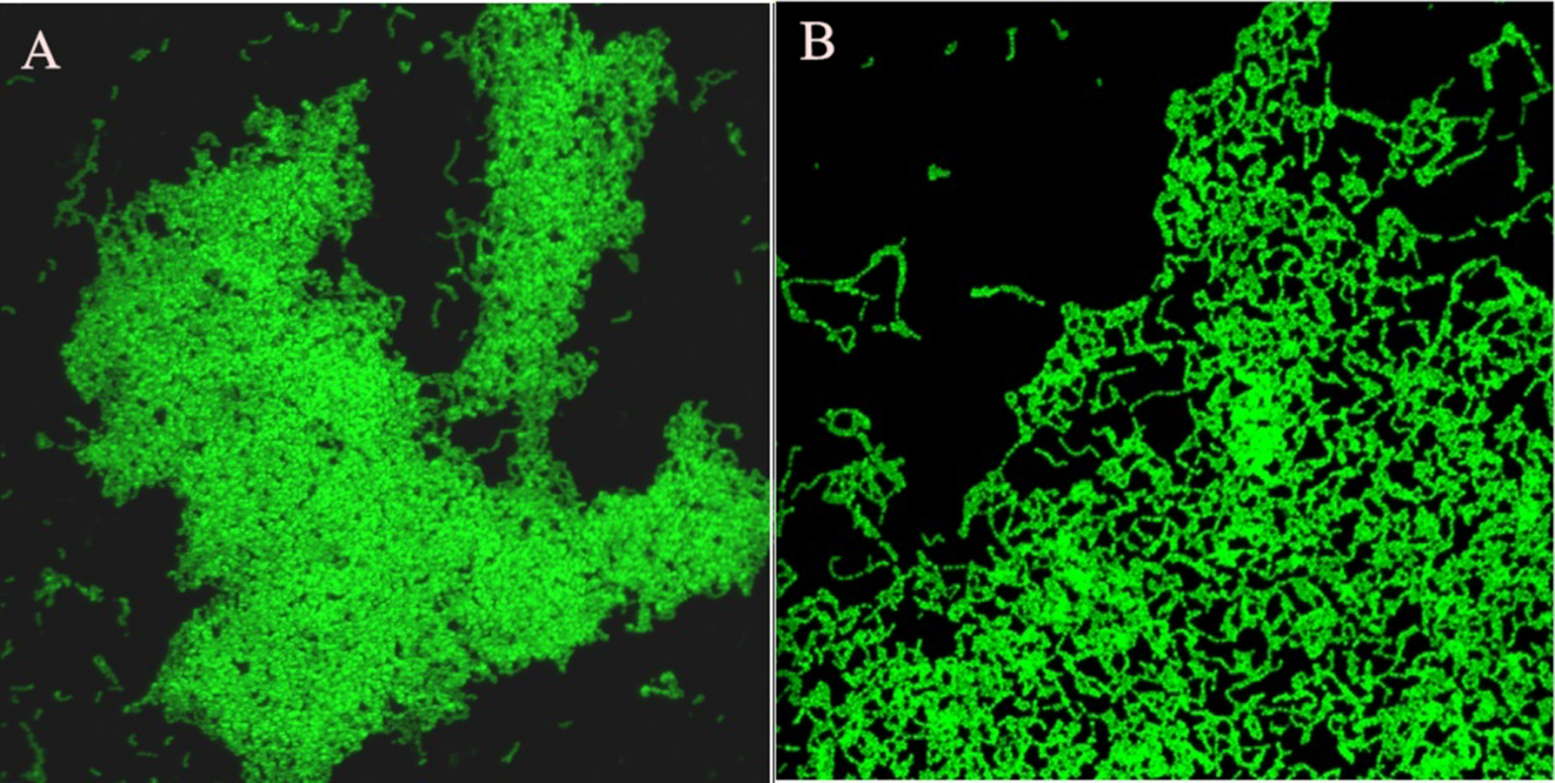
Biofilm analysis using confocal laser scanning microscopy. (A) Representative image of biofilm formed by *S. mutans* ATCC25175, (B) biofilm in the presence of dextranase.

Confocal Laser Scanning Microscopy (CLSM) is a popular method to study biofilm structure. It was carried out to confirm whether the synergized effect of dextranase activity can disrupt matured biofilm produced by *S. mutans* ATCC 25175. The 24 h matured biofilm was exposed to the crude fungal dextranase from *P. roquefortii* TISTR 3511, and the anti-biofilm activity was analyzed by CLSM after staining with SYTO^®^9 green fluorescents. The staining is used to indicate cell viability, as determined by the integrity of the cellular components, including biofilm on the basis of the amount of biofilm formation (Banu et al., 2017; Cerca et al., 2012). The dextranase showed significant reduction in biofilm formation compared with the control with no addition of dextranase, which showed the presence of a dense biofilm produced by *S. mutans* ATCC 25175 (Fig. 1).

### The study of factors affecting encapsulation to achieve a suitable condition

**Figure 2:**
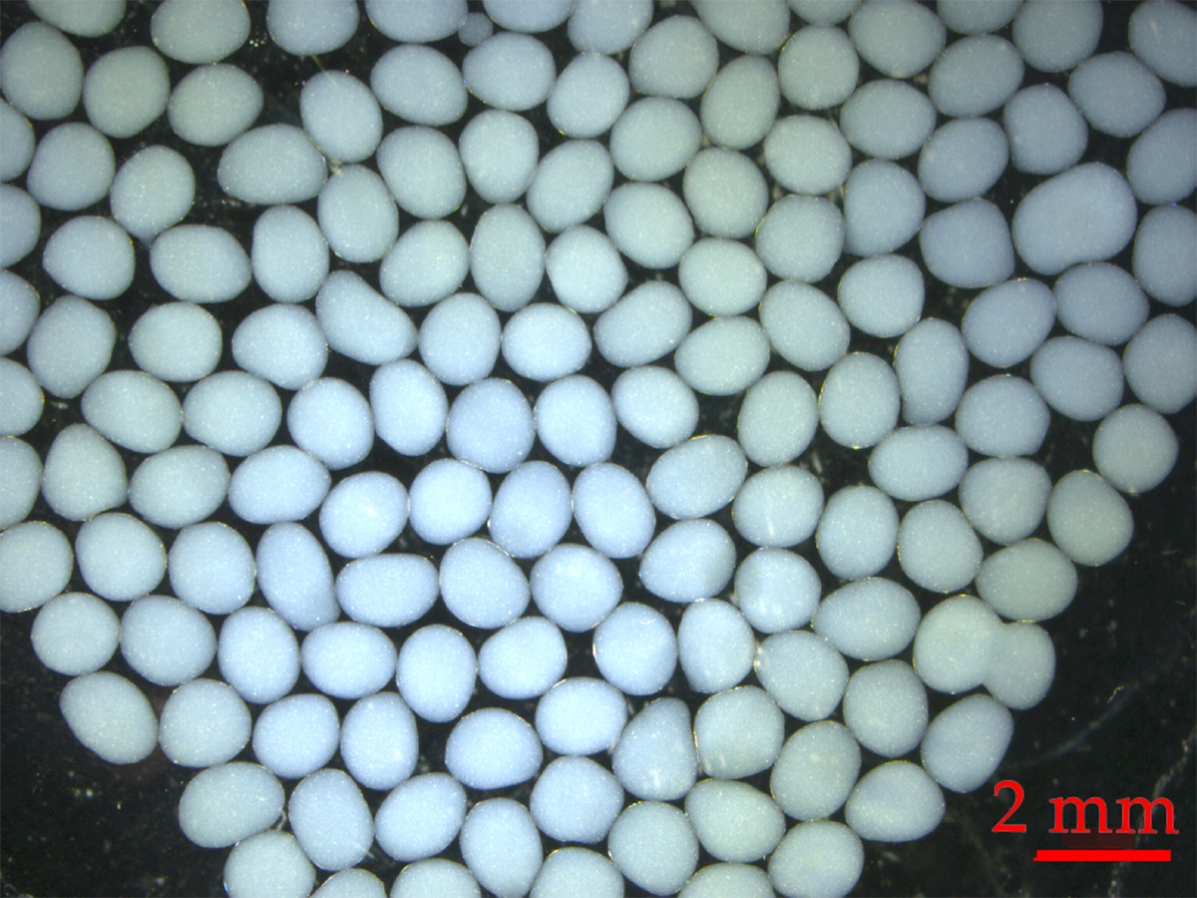
The encapsulation of crude dextranase in alginate beads.

The encapsulation conditions of dextranase enzymes in alginate beads were designed by BBD with 17 conditions of the 3 factors affecting encapsulation: pH, calcium chloride concentration and the concentration of sodium alginate (Table 2; Dataset S2). It was found that Condition No. 14 produced the highest efficiency of enzyme storage (% EE) and enzyme activity. The optimum condition was pH equal to 7, 20% w/v calcium chloride and 0.85% w/v sodium alginate, with the highest activity (18.32 unit/g.bead). Condition No. 14, the most suitable condition for encapsulation, was due to the pH value of 7, which is close to 6, the optimal pH for dextranase. Moreover, the beads from condition No. 14 were regular spherical shape, and the mean diameter of 30 beads was 1.18 ± 0.02 mm (Fig. 2; Table S5).

**Table 2:** The use of Box Behnken design to investigate the suitable condition for encapsulation of commercial fungal dextranase enzyme. The asterisk (*) indicated the condition that produced the highest efficiency of enzyme storage (% EE) and enzyme activity.

**Condition**	**Factor**			**Actual value**		**Predicted value**	
	**pH**	**CaCl**_**2**_**(%w/v)**	**Sodium Alginate(%w/v)**	**%EE(%)**	**Enzyme activity(unit/g.bead)**	**%EE(%)**	**Enzyme activity(unit/g.bead)**
1	6.00	15.00	0.85	40.02	8.39	39.55	8.25
2	6.00	15.00	0.85	31.46	6.55	39.55	8.25
3	6.00	20.00	0.70	24.62	5.08	26.80	5.42
4	6.00	15.00	0.85	46.38	9.76	39.55	8.25
5	6.00	10.00	0.70	8.95	1.71	15.34	3.06
6	6.00	15.00	0.85	45.96	9.67	39.55	8.25
7	7.00	10.00	0.85	73.4	15.57	62.27	13.10
8	5.00	10.00	0.85	52.12	10.78	59.04	12.25
9	5.00	15.00	0.70	33.91	6.86	20.61	4.05
10	7.00	15.00	0.70	42.75	8.76	47.50	9.89
11	6.00	10.00	1.00	33.91	6.86	31.74	6.34
12	6.00	20.00	1.00	70.96	14.83	64.58	13.48
13	7.00	15.00	1.00	33.45	6.76	46.76	9.57
14^∗^	7.00	20.00	0.85	87.17	18.32	80.25	16.85
15	5.00	20.00	0.85	74.23	15.53	85.36	17.99
16	6.00	15.00	0.85	33.91	6.86	39.55	8.25
17	5.00	15.00	1.00	80.27	16.83	75.53	15.70

### The efficiency of encapsulation (% EE)

Based on experimental results (actual) and predicted values, Condition No. 14 showed the highest efficiency of enzyme storage (% EE) and enzyme activity, as previously explained. The maximum value of the efficacy of encapsulation (% EE) was equal to 87.17. Quadratic models are the most suitable models for describing the trend of efficiency of encapsulation in the beads (*p* = 0.0215). The factors that had effect on the efficiency of enzyme storage in the beads showed statistical significance, such as sodium alginate concentration (*p* = 0.0156) and calcium chloride concentration (*p* = 0.0356), as shown in Table 3 and Dataset S2.

The *F*-value of 5.10 showed that this model was significant at a *p*-value of 0.0215. The lack of fit, *F*-value of 6.00 and *p*-value of 0.0581 implied unimportance due to relative pure error. The values of *R*^2^ and adjusted *R*^2^ were found to be 0.8677 and 0.6977 respectively, while the lack of significance of the value (*p* = 0.0581) of lack of fit indicates that the model was fitted with good prediction (Singh, Bajar & Bishnoi, 2017).

**Table 3:** The statistical analysis of factors affecting the efficiency of commercial fangal dextranase encapsulation.

**Source**	**Sum ofSquares**	**df**	**MeanSquare**	**FValue**	**p-valueProb >F**
Quadratic Model	6684.17	9	742.69	5.10	0.0215*
A- Conc. Alginate	1467.74	1	1467.74	10.08	0.0156*
B-Conc. CaCl_2_	981.24	1	981.24	6.74	0.0356*
C- pH	1.77	1	1.77	0.012	0.9154
AB	114.28	1	114.28	0.79	0.4050
AC	774.51	1	774.51	5.32	0.0544
BC	17.39	1	17.39	0.12	0.7398
A^2^	889.60	1	889.60	6.11	0.0427
B^2^	388.00	1	388.00	2.67	0.1465
C^2^	2147.62	1	2147.62	14.76	0.0064
Residual	1018.82	7	145.55	–	–
Lack of Fit	833.60	3	277.87	6.00	0.0581
Pure Error	185.22	4	46.30	–	–
Corrected Total	7702.99	16	–	–	–

From the above experiment, the relationship between factors and responses (% EE) values can be expressed using regression as shown in the following equations. Letters A, B and C are alginate, calcium chloride and pH respectively.

\begin{eqnarray*}\text{%}~EE=39.546+13.545A+11.075B-0.470C+5.345AB-13.915AC-2.085BC\nonumber\\\displaystyle  -14.536AA+9.600BB+22.584CC \end{eqnarray*}

**Figure 3:**
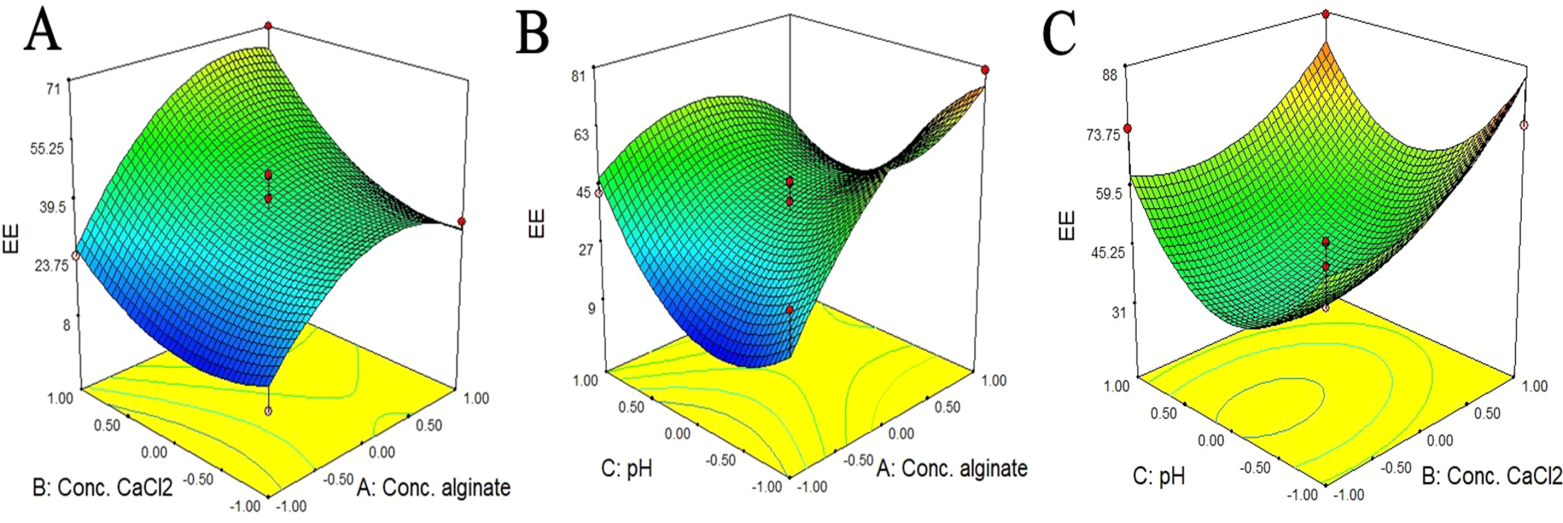
Response surface for the interaction of the independent variables in relation to the efficiency of enzyme storage (% EE). (A) Effect of calcium chloride concentration (%w/v) and sodium alginate concentration (%w/v); (B) effect of pH and sodium alginate concentration (%w/v); (C) effect of pH and calcium chloride concentration (%w/v).

A response surface plot can be used to analyze the interaction between two factors in relation to the efficiency of enzyme storage (% EE). The shape of a contour plot reflects the intensity of the interaction between the factors to a certain extent (Zhang et al., 2017). The responses are shown in Fig. 3, where red and blue are the maximum and minimum encapsulation efficiencies, respectively.

The interaction between the calcium chloride concentration (%w/v) and sodium alginate concentration (% w/v) (Fig. 3A) was indicated by the *p* value = 0.4050, while the interaction between pH and sodium alginate concentration (% w/v) (Fig. 3B) was indicated by the *p* value = 0.0544. For the interaction between pH and calcium chloride concentration (% w/v) (Fig. 3C), it was indicated by the *p* value = 0.7398. The interactions (Figs. 3A–3C) showed no significance; however, the optimum condition was considered as the maximum encapsulation efficiency, which was found at pH of 7, 20% w/v calcium chloride and 0.85% w/v sodium alginate.

### The activity of dextranase enzyme encapsulated in alginate beads

Condition No. 14 also produced the highest enzyme activity, which was 18.32 unit /g. bead. The statistical analysis of the activity of the enzyme trapped in the alginate beads for all 17 conditions found that the quadratic model was the most suitable model for describing the activity of encapsulated enzyme, *p* = 0.0236. The factors that were statistically significant for effect on enzyme activity were the concentrations of calcium chloride and alginate, as shown in Table 4 and Dataset S2.

The *F*-value of 4.93 showed that this model was significant at a *p*-value of 0.0236. The lack of fit, *F*-value of 5.66 and *p*-value of 0.0637 implied unimportance due to relative pure error. In addition, the values of *R*^2^ and adjusted *R*^2^ were found to be 0.8637 and 0.6885 respectively, while the lack of significance of the value (*p* = 0.0637) of lack of fit indicates that the model was fitted with good prediction (Singh, Bajar & Bishnoi, 2017).

From the above experiment, the relationship between factors and responses (dextranase activity) values can be expressed using regression, as shown in the following equations. Letters A, B and C are alginate, calcium chloride and pH respectively.

\begin{eqnarray*}Dextranase~activity=8.246+2.836A+2.376B-0.073C+1.195AB-2.994AC-0.498BC\nonumber\\\displaystyle  -3.208AA+2.037BB+4.765CC \end{eqnarray*}

**Figure 4:**
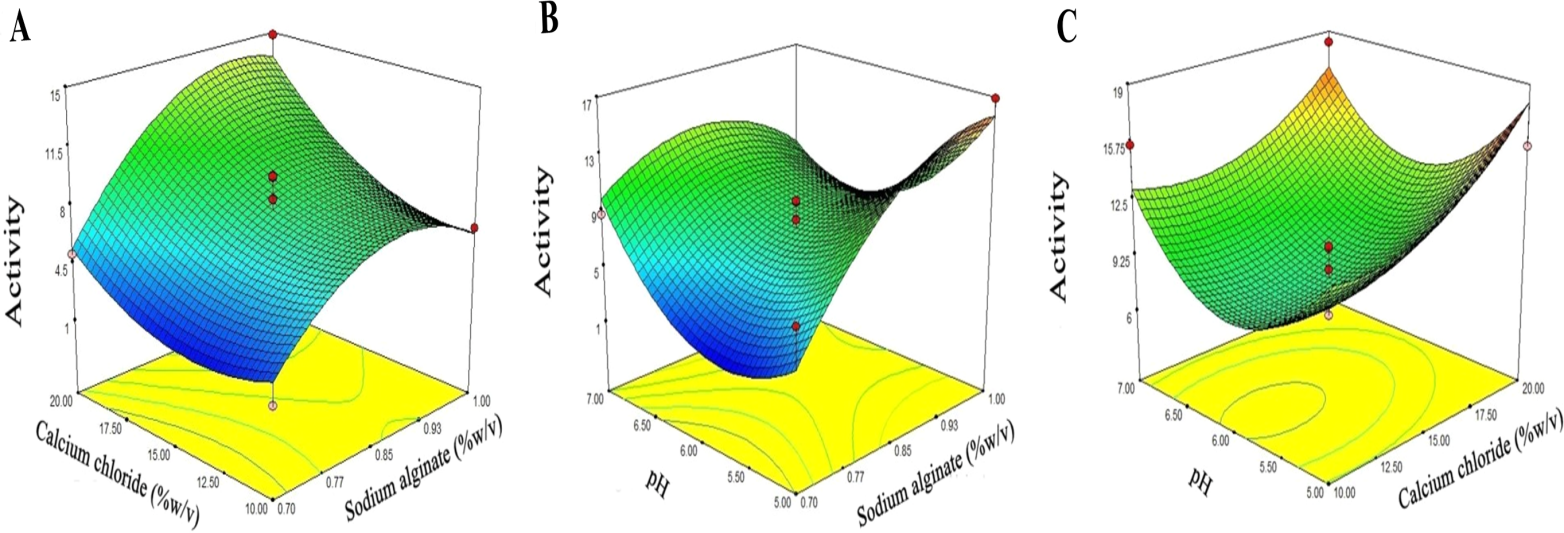
Response surface for the interaction of the independent variables in relation to the efficiency of dextranase activity. (A) Effect of calcium chloride concentration (% w/v) and sodium alginate concentration (% w/v); (B) effect of pH and sodium alginate concentration (% w/v); (C) effect of pH and calcium chloride concentration (% w/v).

Figures 4A–4C shows the response surface plots for the interactions between various factors, where red and blue are the maximum and minimum dextranase enzyme activities of encapsulation respectively. In addition, the interaction between the calcium chloride concentration (% w/v) and sodium alginate concentration (% w/v) (Fig. 4A) was indicated by the *p*-value = 0.3913. The interaction between pH and sodium alginate concentration (% w/v) (Fig. 4B) was indicated by the *p*-value = 0.0560, and the interaction between pH and calcium chloride concentration (% w/v) (Fig. 4C) was indicated by the *p*-value = 0.7150. The interactions between various factors (Figs. 4A–4C) showed no significance.

**Table 4:** The statistical analysis of factors that affect dextranase enzyme activity in beads.

**Source**	**Sum ofSquares**	**df**	**MeanSquare**	**FValue**	**p-valueProb >F**
Quadratic Model	303.69	9	33.74	4.93	0.0236*
A-Conc. Alginate	64.35	1	64.35	9.40	0.0182*
B-Conc. CaCl_2_	45.17	1	45.17	6.60	0.0371*
C-pH	0.042	1	0.042	0.01	0.9397
AB	5.71	1	5.71	0.83	0.3913
AC	35.82	1	35.82	5.23	0.0560
BC	0.99	1	0.99	0.14	0.7150
A^2^	43.33	1	43.33	6.33	0.0400*
B^2^	17.47	1	17.47	2.55	0.1541
C^2^	95.58	1	95.58	13.96	0.0073*
Residual	47.91	7	6.84	–	–
Lack of Fit	38.77	3	12.92	5.66	0.0637
Pure Error	9.14	4	2.28	–	–
Corrected Total	351.60	16	–	–	–

Considering the two responses (encapsulation efficiency and trapped dextranase activity in alginate beads), it was found that the optimum condition was the same as a pH equal to 7, 20% w/v calcium chloride concentration and 0.85% w/v sodium alginate concentration. Therefore, this condition was selected for producing dextranase beads for further study of the stability of the enzyme in oral care product (toothpaste).

### The stability of encapsulated dextranase activity in a toothpaste base

A 2% alginate beads containing dextranase enzyme was investigated to determine the stability of dextranase activity in toothpaste formulation at baseline and after 3 months of testing. The data was analyzed by the *t*-test statistic, and it was found that the dextranase activity in the beads was not significantly different (*p*-value = 0.1423) after storage of the product at 40 ± 2 °C for 3 months (Table 5; Table S6). In addition, the characteristics of the toothpaste did not change after 3 months compared to baseline. Generally, the stability of the toothpaste was still according to the properties of good toothpaste, i.e., no change of appearance based on color, odor, viscosity, pH and homogeneous.

### The satisfaction of toothpaste

The volunteers were selected with inclusion and exclusion criteria. The experiment was conducted under the supervision of an expert dentist. The demographic data of volunteers indicated that there were 15 volunteers, 5 males and 10 females. The average age was 35 years, and all the volunteers did not have a wound in the oral cavity. Then, they were given brush and toothpaste containing beads with commercial dextranase enzyme to brush once for 2 min for assessment, and their oral cavities were evaluated by the dentist again. The result showed that they did not have irritation after brushing.

**Figure 5:**
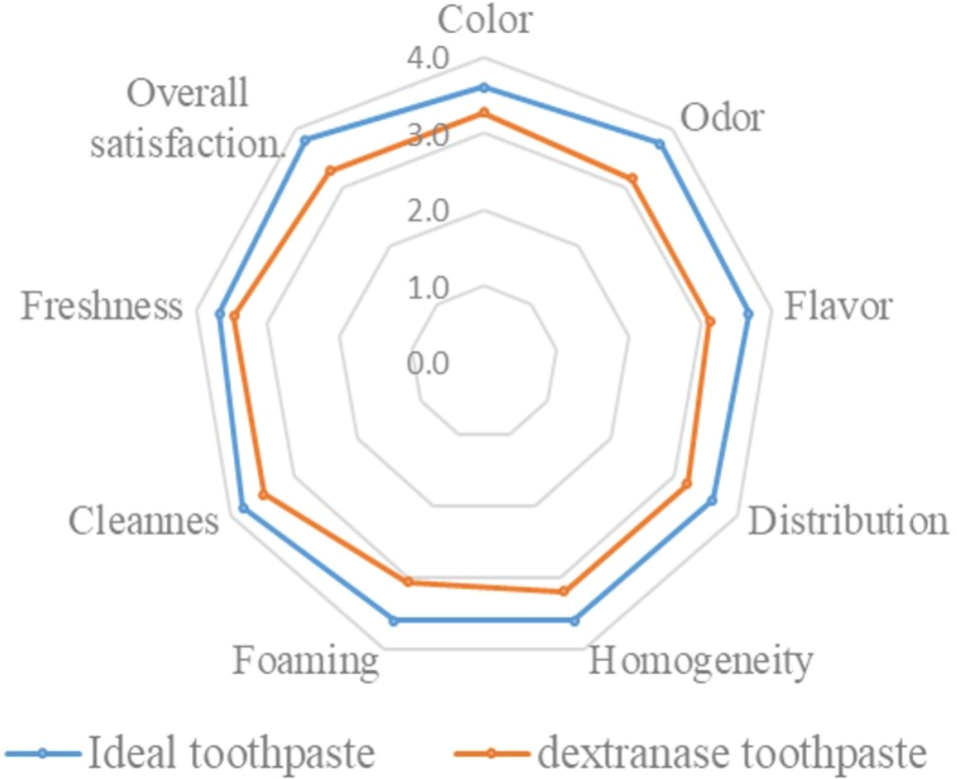
The sensory satisfaction of the toothpaste containing alginate beads with dextranase enzyme by 15 volunteers.

Regarding the sensory satisfaction of the toothpaste containing alginate beads with dextranase enzyme, most volunteers chose “like slightly” (3 points) for all parameters: color, odor, flavor, the distribution of toothpaste, homogeneity of ingredient, the amount of toothpaste foaming while brushing, freshness, cleanness and overall satisfaction (Fig. 5; Table S7). The volunteers preferred the ideal toothpaste (near 4 points).

**Table 5:** The activity of commercial fungal dextranase activity in alginate beads of toothpaste.

**Treatment**	**Dextranase enzyme activity(unit/g.breads)**		***p*-value**
	**T0**	**T3**	
Toothpaste base containing 2% w/w beads with commercial dextranase	1775.40 ± 21.82	1615.83 ± 95.66	0.1423

**Notes.**

T0 = day 0, T3 = Storage product at 40 ± 2 °C for 3 months.

## Discussion

Dental caries is tooth decay caused by bacteria in the oral cavity by the process of demineralization. Caries begins with dental plaque formation on teeth surfaces. *S. mutans* is the most common cariogenic bacterial species isolated from human dental plaque. Currently, there are many active ingredients used in different formulations to prevent tooth decay. In order to enhance the natural antimicrobial defense mechanisms, oral health products, including toothpastes, with different added ingredients have been used (Pedersen et al., 2019). The use of metabolites from microorganisms for oral care is an emerging trend in the world. Dextranase can prevent dental caries by inhibiting the adherence of *S. mutans* to teeth and reducing the biofilm formed once adhered via water-insoluble glucan. Dextranase may reduce the formation and accumulation of *S. mutans* biofilm by diminishing water-soluble glucan in the matrix (Wang et al., 2014). The use of topical dextranase as an approach to control dental plaque has been explored by both in vitro and in vivo studies with different results.

This research focused on the application of microbial metabolites, like fungal dextranase enzyme, in oral care, which is an emerging trend in the world. Dextran is a branched polysaccharide and one of the polymers present in biofilm matrix. It plays a perilous role in dental plaque formation and is involved in the development of some common oral diseases, like dental caries. Dextran-hydrolyzing enzymes are under investigation to treat and manage dental plaques. Enzymatic hydrolysis of dextran is carried out with the enzyme dextranase, which breaks down the polysaccharide dextran to smaller oligosaccharide molecules. The mechanisms for preventing oral diseases are the elimination of *S. mutans* and plaque removal. The highest activity of the commercial fungal dextranase used in this study occurred at a pH of 6 and 37 °C. Generally, the highest activity depends on the species of microorganisms. For instance, a novel purified dextranase from *Penicillium cyclopium* CICC-4022 had the highest activity at 55 °C and a pH of 5.0 (Huang et al., 2019), while the optimal condition for dextranase enzyme produced by *Penicillium funiculosum* was a pH of 4.5–5.0 and 55–60 °C (Volkov et al., 2019). This means that the commercial dextranase produced by *C. gracili*, which is used in the present study, is a mesophilic enzyme, as its activity decreased 4 times at 55 °C compared with 37 °C. It should be noted that the optimal condition of the dextranase (pH of 6–7 and temperature of 37 °C) used in the present study is similar to the condition of the oral cavity (Foglio-Bonda et al., 2017; Saini et al., 2014), so it should be suitable for use in the oral cavity to prevent dental caries.

To apply dextranase in oral care products against the growth of *S. mutans*, the lowest and effective quantity is considered. The cost of production and efficacy are also important. Hence, the MIC and a lower dose than the MIC of dextranase enzyme were investigated. The MIC of the commercial dextranase was found to be 4.5 unit/g, so it is possible to apply this enzyme in toothpaste.The dextranase enzyme can reduce the adherence of *S. mutans* to teeth surfaces by the degradation of biofilm (Kim, Sung & Kim, 2013); it acts against cariogenic microorganisms, particularly *S. mutans*, to prevent dental caries and dental plaque. To confirm the efficacy of dextranase enzyme in biofilm removal, which is one of the ways to prevent dental plaque, the growth of *S. mutans* in the presence of dextranase enzyme at sub-MICs was compared with its growth in the absence of dextranase. Normally, bacteria still continue to grow at sub-MIC (Sato et al., 2018). The experiments performed in our study enabled us to measure the rate of adherence and subsequent biofilm formation of a tested bacterium, *S. mutants* ATCC 25175. Dextranase can inhibit the synthesis of insoluble biofilm as well as the adherence of streptococci (Khalikova, Susi & Korpela, 2005). The initial interest in dextranases was raised with regard to their possible application in commercial production. Dextranase at 1/2 MIC had the ability to reduce biofilm formation by *S. mutans* ATCC 25175 to weak adherence compared with the strong adherence of this bacterium (Table 1). This suggests the possibility of using this commercial fungal dextranase in toothpaste. However, the stability of the enzyme is very important in its application; therefore, encapsulation using alginate was employed by investigating the optimal condition to protect the stability of the enzyme.

The encapsulation of enzymes is an active topic of research in enzyme technology and is essential for the application of such systems to industrial processes (Blandino, Macías & Cantero, 2003). A previous research showed that enzymes were co-immobilized by encapsulating soluble dextransucrase and dextranase covalently attached in alginate. The alginate capsule resulted in a high immobilization yield (71%), and enzymatic activities were without any decrease after a month in storage at 4 °C (Ölçer & Tanriseven, 2010). This suggests that encapsulation enhances the stability of those enzymes for industrial-scale production. Regarding the present study, encapsulation of commercial fungal dextranase in alginate beads under optimal condition (pH of 7, 0.85% sodium alginate and 20% CaCl_2_) showed maximum % EE of 87.17% with the maximum dextranase activity at 18.32 unit/ g.beads (Table 2). This indicates the potential of encapsulated dextranase in alginate beads to be used in toothpaste. To increase the efficiency of encapsulation along with good stability and well release while brushing teeth, it would be worthwhile to study with other biopolymers or blending alginate with starch. This was due to the fact that some loss of active compounds occurs during preparation of alginate beads, while adding starch into alginate improves encapsulation efficiency (Khlibsuwan, Tansena & Pongjanyakul, 2018).

The use of antimicrobials as an important adjunct to tooth brushing may also play a role in controlling biofilm. Also, the incorporation of chemical agents with antimicrobial activity into dental products has been proposed as a potential way of reducing plaque-mediated disease (Amižić et al., 2017). The possible action of toothpaste containing dextranase enzyme in the oral environment is the digestion of biofilm. This is a relatively new concept in the prevention of oral infectious diseases, such as caries. The dextranase encapsulated in beads is released while brushing as the force and speed are the principles of mechanical to break the beads (Cooper, 2013). Thus, during brushing the active compound is released and contacting with the tooth surface. While the anti-biofilm agent like dextranase remains active in the oral cavity for a prolonged period of time after brushing (Otten et al., 2012). The dextranase contacts with the plaque as its substrate to the same extent like other active compounds in toothpaste commercials for preventing caries. Hence, dextranase may prevent dental caries by the degradation of dextran, which is a component of plaque biofilm.

Normally, brushing is the most suitable mechanical method for reducing biofilm, while toothpaste increases the efficiency of cleaning the mouth even more. Toothpaste is a personal care product that people use every day for oral freshness and/or other functions. Chlorhexidine is considered to be the “gold standard” antiplaque in oral care products due to its prolonged broad-spectrum antimicrobial activity and plaque inhibitory potential. However, even when using chlorhexidine as directed, a bitter aftertaste might be noticed and can last for several hours, and people can have adverse effects and allergic reactions even with normal use (Prasanth, 2011). In contrast, the dextranase hydrolyzes the *α*-(1-6)-d-glycoside linkages in dextran that is the composition of oral plaque structure. The results of present study proved that dextranase acting not only to reduce biofilm or plaque but also to inhibit the growth of *S. mutans* as biofilm producer. It should be noted that the volunteers did not have irritation after brushing compared with toothpaste containing chlorhexidine (Prasanth, 2011).

The purpose of cosmetic product stability testing is to ensure that a new or modified product meets the intended physical, chemical and microbiological quality standards as well as the intended functionality and aesthetics when stored under appropriate conditions (CTFA & COLIPA, 2004). Thus, this research focused on encapsulation to increase the stability of dextranase activity in toothpaste. The results showed that the dextranase activity of the encapsulated dextranase in toothpaste was stable after storage at 40 ± 2 °C for 3 months. These results indicate that encapsulation could be a method of improving the dextranase activity in toothpaste. However, the shelf life of the product must be evaluated as well.

Most of the toothpaste-related studies have focused on the functionality of ingredients (Lippert, 2013). However, from the consumer point of view, functional ingredients affecting dental whitening or having anti-cavity property do not seem to adequately draw consumers’ interest because competition in the toothpaste market has become very intense. Instead, flavors seem to be a dominant factor of interest because it is the main characteristic that people easily notice during tooth brushing. Westerink & Kozlov (2004) suggested that oral freshness, which occurs during tooth brushing or mouth cleaning, gives possible rewards. The freshness of toothpastes comes from flavor-related ingredients, such as menthol. Toothpastes have various flavor characteristics, but there are few sensory-related toothpaste studies, especially those focused on flavor (Kim, Sung & Kim, 2013). The color odor viscosity and texture properties of toothpaste are very important parameters that influence production and customer satisfaction (Toksoy, Tugcu-Demiröz & Tirnaksiz, 2013). Therefore, this study conducted tests to evaluate the product satisfaction and safety. This research found satisfaction of developed toothpaste was lower than commercial toothpaste. It could be possible to achieve higher satisfaction by improvement both smell and taste of the product. Hence, the sensory satisfaction of the developed toothpaste has to be developed to achieve the goal as the ideal toothpaste.

In this study, product risk assessments for volunteers were done initially to make sure that toothpaste containing encapsulated commercial fungal dextranase in alginate beads was not dangerous to them. The experiment was conducted under the supervision of an expert dentist. All the volunteers did not have irritation after brushing, indicating the safety of the developed toothpaste. Moreover, the suitable size of the beads and enzyme quantity for packing in toothpaste should be studied in human volunteers after brushing to have the enzyme residual sufficient for the function and efficiency in reducing biofilm. Moreover, this research should further study in humans to confirm efficacy of encapsulated dextranase beads by looking at the plaque index (PI), before and after at least 1 month after using the developed toothpaste.

## Conclusions

The commercial dextranase produced by *C. gracili* is a mesophilic enzyme, and it showed anti-*S. mutans* growth and anti-biofilm formation, which are the main mechanisms to prevent dental caries; its effective dose is 4.5 unit/g. The use of encapsulation under the optimal condition of a pH of 7, 0.85% sodium alginate and 20% CaCl_2_ achieved 87.17% EE with dextranase activity of 18.32 unit/g bead. This allows encapsulated dextranase in 2% alginate beads to be applied in toothpaste as it showed stability for 3 months. The developed toothpaste containing encapsulated dextranase alginate beads meets the criteria of safety and like slightly for its sensory test. This research explored an alternative health toothpaste product for oral care.

##  Supplemental Information

10.7717/peerj.10165/supp-1 Supplemental Information 1 Physical properties of toothpaste

10.7717/peerj.10165/supp-2 Supplemental Information 2 Dextranase activity

10.7717/peerj.10165/supp-3 Supplemental Information 3 MIC test

10.7717/peerj.10165/supp-4 Supplemental Information 4 Anti-biofilm

10.7717/peerj.10165/supp-5 Supplemental Information 5 Beads size

10.7717/peerj.10165/supp-6 Supplemental Information 6 Stability

10.7717/peerj.10165/supp-7 Supplemental Information 7 Satisfaction

10.7717/peerj.10165/supp-8 Supplemental Information 8 Plackett Burman design

10.7717/peerj.10165/supp-9 Supplemental Information 9 Box Behnken design

## Additional Information and Declarations

### Data Availability

The following information was supplied regarding data availability: The raw data are available in the Supplemental Files.
