# Supplementary figures and images for: Anti-Streptococcus mutans and anti-biofilm activities of dextranase and its encapsulation in alginate beads for application in toothpaste
Source: PeerJ. 2020 Nov 17;8:e10165. doi: 10.7717/peerj.10165 (PMC7678491; doi:10.7717/peerj.10165)

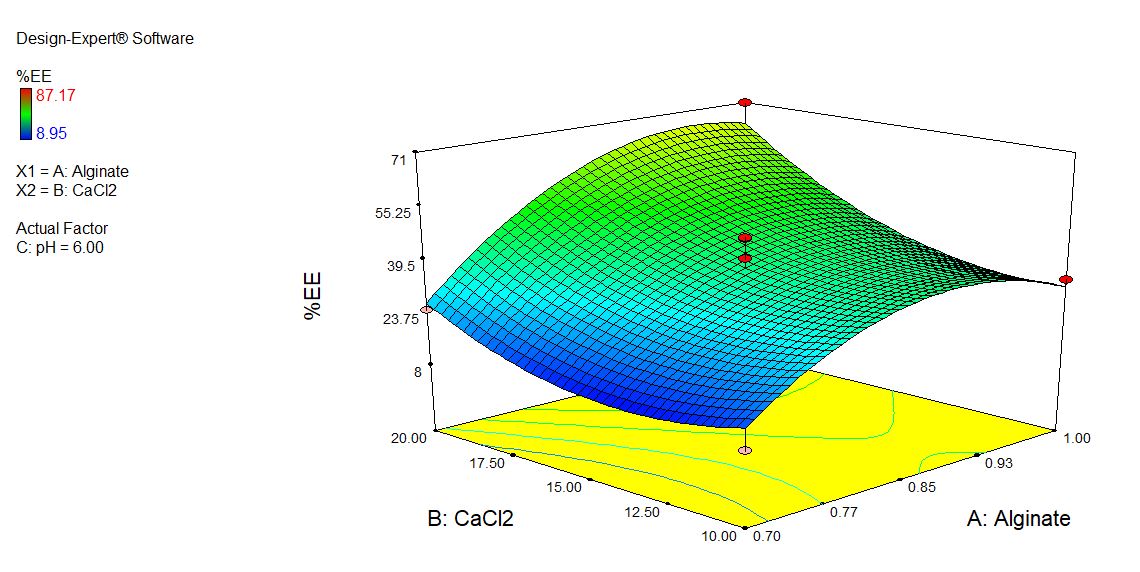

Supplement: Supplemental Information 9 [file peerj-08-10165-s009.zip › Dataset S2/%EE/%EE-CaCl2-Alginate.JPG]

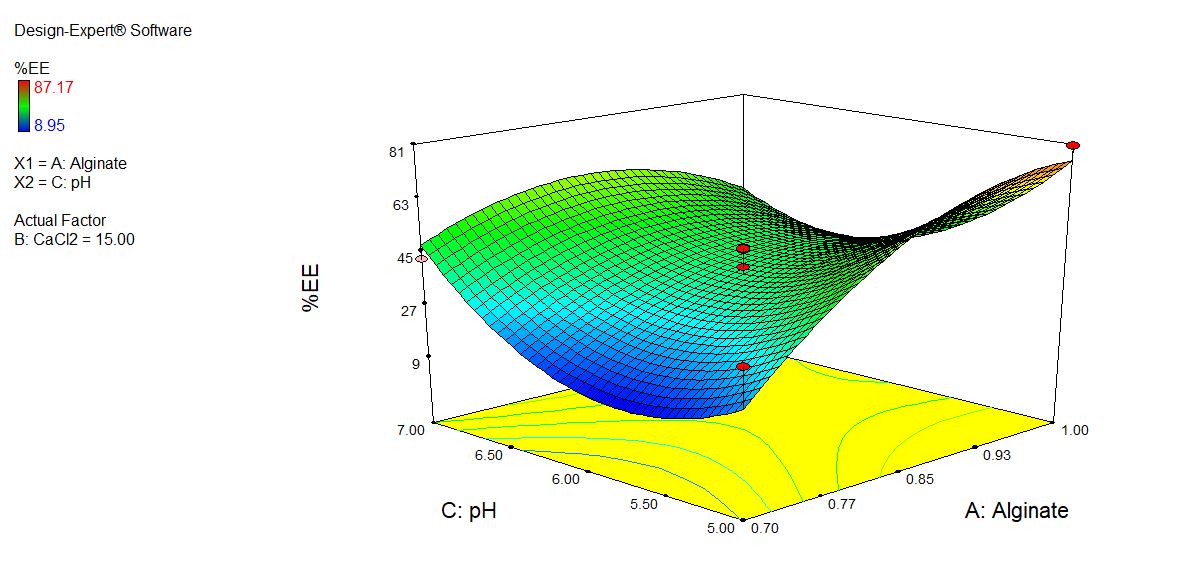

Supplement: Supplemental Information 9 [file peerj-08-10165-s009.zip › Dataset S2/%EE/%EE-pH-Alginate.JPG]

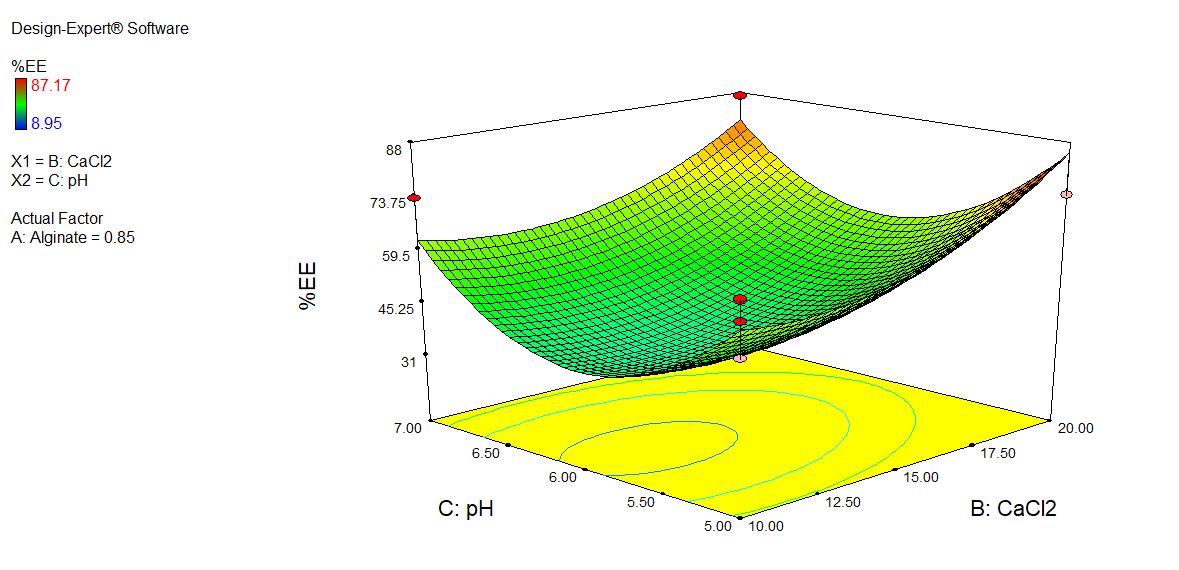

Supplement: Supplemental Information 9 [file peerj-08-10165-s009.zip › Dataset S2/%EE/%EE-pH-CaCl2.JPG]

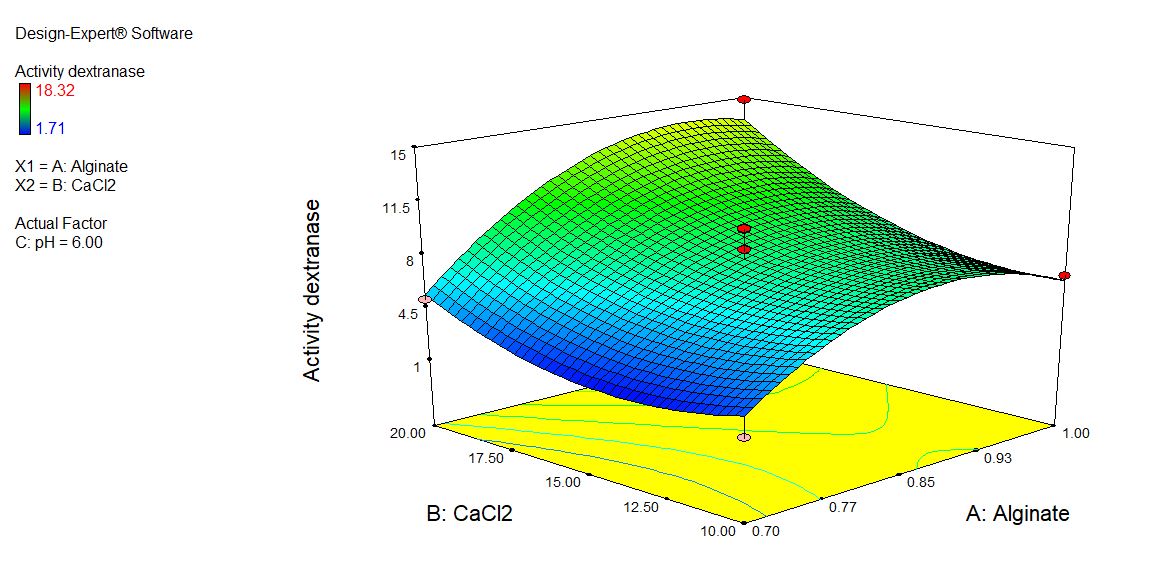

Supplement: Supplemental Information 9 [file peerj-08-10165-s009.zip › Dataset S2/Dextranase activity/Activity-CaCl2-Alginate.JPG]

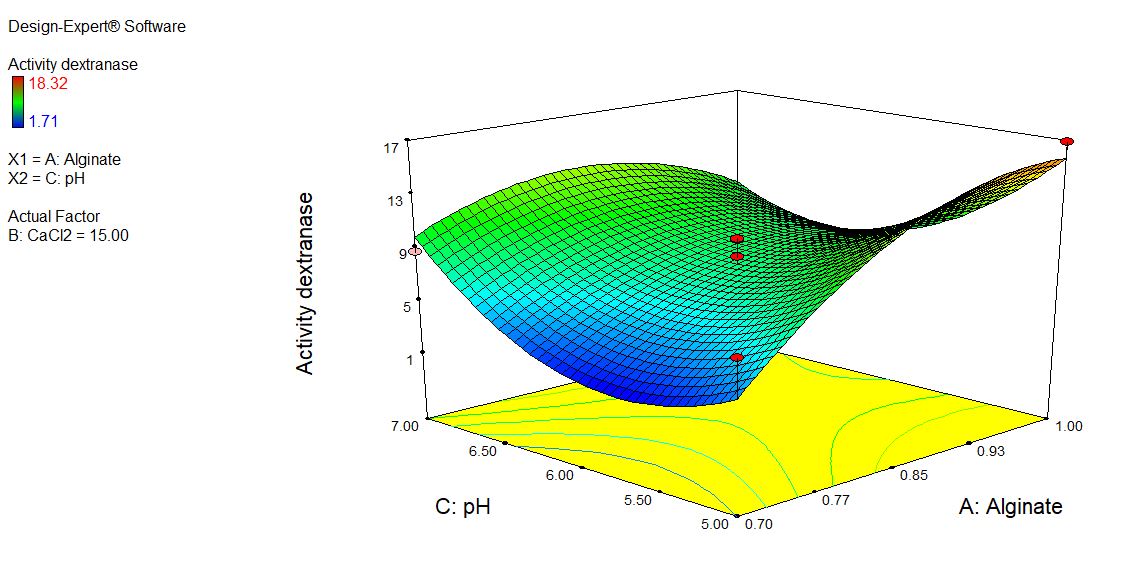

Supplement: Supplemental Information 9 [file peerj-08-10165-s009.zip › Dataset S2/Dextranase activity/Activity-pH-Alginate.JPG]

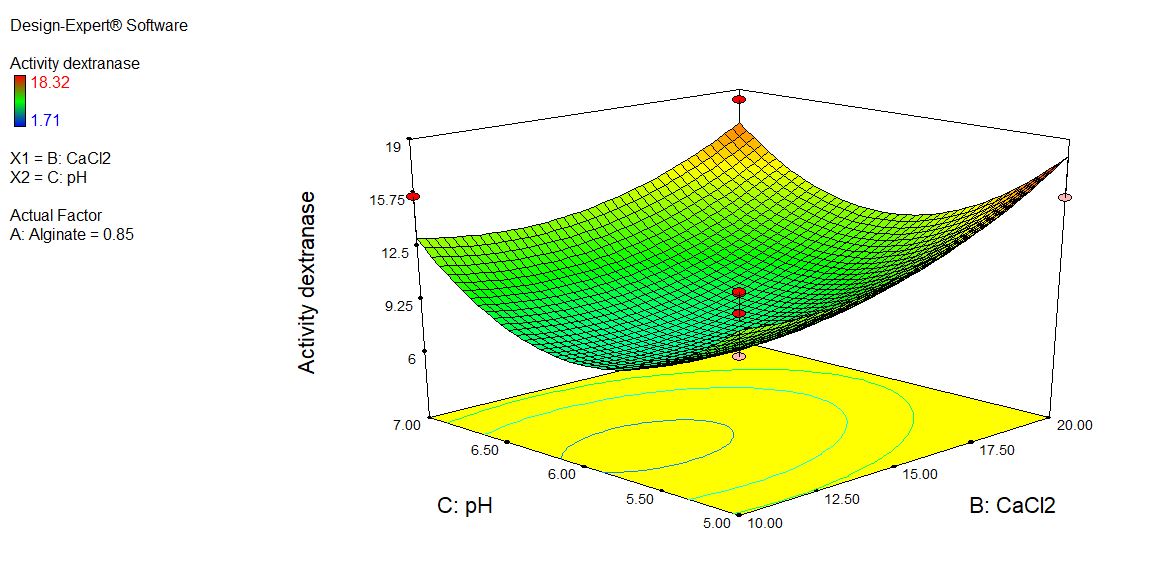

Supplement: Supplemental Information 9 [file peerj-08-10165-s009.zip › Dataset S2/Dextranase activity/Activity-pH-CaCl2.JPG]
